# Time, pattern, and outcome of medulloblastoma relapse and their association with tumour biology at diagnosis and therapy: a multicentre cohort study

**DOI:** 10.1016/S2352-4642(20)30246-7

**Published:** 2020-12

**Authors:** Rebecca M Hill, Stacey Richardson, Edward C Schwalbe, Debbie Hicks, Janet C Lindsey, Stephen Crosier, Gholamreza Rafiee, Yura Grabovska, Stephen B Wharton, Thomas S Jacques, Antony Michalski, Abhijit Joshi, Barry Pizer, Daniel Williamson, Simon Bailey, Steven C Clifford

**Affiliations:** a Wolfson Childhood Cancer Research Centre, Newcastle University Centre for Cancer, Newcastle upon Tyne, UK; b Department of Applied Sciences, Northumbria University, Newcastle upon Tyne, UK; c School of Electronics, Electrical Engineering and Computer Science, Queen's University Belfast, Centre for Cancer Research & Cell Biology, UK; d Sheffield Institute for Translational Neuroscience, University of Sheffield, Sheffield, UK; e Neural Development Unit, UCL Institute of Child Health, London, UK; f Department of Neuropathology, Royal Victoria Infirmary, Newcastle University Teaching Hospitals, NHS Foundation Trust, Newcastle upon Tyne, UK; g Institute of Translational Research, University of Liverpool, Liverpool, UK

## Abstract

**Background:**

Disease relapse occurs in around 30% of children with medulloblastoma, and is almost universally fatal. We aimed to establish whether the clinical and molecular characteristics of the disease at diagnosis are associated with the nature of relapse and subsequent disease course, and whether these associations could inform clinical management.

**Methods:**

In this multicentre cohort study we comprehensively surveyed the clinical features of medulloblastoma relapse (time to relapse, pattern of relapse, time from relapse to death, and overall outcome) in centrally reviewed patients who relapsed following standard upfront therapies, from 16 UK Children's Cancer and Leukaemia Group institutions and four collaborating centres. We compared these relapse-associated features with clinical and molecular features at diagnosis, including established and recently described molecular features, prognostic factors, and treatment at diagnosis and relapse.

**Findings:**

247 patients (175 [71%] boys and 72 [29%] girls) with medulloblastoma relapse (median year of diagnosis 2000 [IQR 1995–2006]) were included in this study. 17 patients were later excluded from further analyses because they did not meet the age and treatment criteria for inclusion. Patients who received upfront craniospinal irradiation (irradiated group; 178 [72%] patients) had a more prolonged time to relapse compared with patients who did not receive upfront craniospinal irradiation (non-irradiated group; 52 [21%] patients; p<0·0001). In the non-irradiated group, craniospinal irradiation at relapse (hazard ratio [HR] 0·27, 95% CI 0·11–0·68) and desmoplastic/nodular histology (0·23, 0·07–0·77) were associated with prolonged time to death after relapse, *MYC* amplification was associated with a reduced overall survival (23·52, 4·85–114·05), and re-resection at relapse was associated with longer overall survival (0·17, 0·05–0·57). In the irradiated group, patients with MB_Group3_ tumours relapsed significantly more quickly than did patients with MB_Group4_ tumours (median 1·34 [0·99–1·89] years *vs* 2·04 [1·39–3·42 years; p=0·0043). Distant disease was prevalent in patients with MB_Group3_ (23 [92%] of 25 patients) and MB_Group4_ (56 [90%] of 62 patients) tumour relapses. Patients with distantly-relapsed MB_Group3_ and MB_Group4_ displayed both nodular and diffuse patterns of disease whereas isolated nodular relapses were rare in distantly-relapsed MB_SHH_ (1 [8%] of 12 distantly-relapsed MB_SHH_ were nodular alone compared with 26 [34%] of 77 distantly-relapsed MB_Group3_ and MB_Group4_). In MB_Group3_ and MB_Group4_, nodular disease was associated with a prolonged survival after relapse (HR 0·42, 0·21–0·81). Investigation of second-generation MB_Group3_ and MB_Group4_ molecular subtypes refined our understanding of heterogeneous relapse characteristics. Subtype VIII had prolonged time to relapse and subtype II had a rapid time from relapse to death. Subtypes II, III, and VIII developed a significantly higher incidence of distant disease at relapse whereas subtypes V and VII did not (equivalent rates to diagnosis).

**Interpretation:**

This study suggests that the nature and outcome of medulloblastoma relapse are biology and therapy-dependent, providing translational opportunities for improved disease management through biology-directed disease surveillance, post-relapse prognostication, and risk-stratified selection of second-line treatment strategies.

**Funding:**

Cancer Research UK, Action Medical Research, The Tom Grahame Trust, The JGW Patterson Foundation, Star for Harris, The Institute of Child Health - Newcastle University - Institute of Child Health High-Risk Childhood Brain Tumour Network (co-funded by The Brain Tumour Charity, Great Ormond Street Children's Charity, and Children with Cancer UK).

**Research in context**

**Evidence before this study**

Relapse after conventional upfront therapies is the most adverse prognostic factor for childhood medulloblastoma. However, associations between molecular pathology at diagnosis and relapse characteristics are not well understood. We searched PubMed for articles published between Jan 1, 1990, and Dec 31, 2019, with the search terms “medulloblastoma”, and “relapse OR recurrence”. Several clinical and biology studies were identified and reviewed. Two initial reports have highlighted the clinical potential in such studies. The first investigated molecular subgroup at diagnosis in three independent cohorts (n=30, 77, and 96), and delineated subgroup-specific differences in relapse patterns and timings. However, findings showed variability between cohorts, and this study did not consider clinical and molecular features other than subgroup, treatment-related differences, or the radiological patterns of distantly-relapsed tumours. The second reported a retrospective analysis of a European trial for standard-risk medulloblastoma (HIT-SIOP-PNET4; completion 2006), describing in detail the patterns of disease relapse in all patients who relapsed (n=72). However, biological annotation at diagnosis was limited and this trial did not include patients at a high risk of relapse.

**Added value of this study**

Systematic assessment of the nature of medulloblastoma relapse, and its relationship to established and novel clinical and molecular features assessable at diagnosis and treatment received is essential to establish any evidence base for altered clinical practice. We report comprehensive investigation of the molecular, clinical, and radiological features, and the inter-relationships, of a centrally reviewed cohort of 247 children with medulloblastoma, who relapsed after conventional upfront therapies. These are estimated to represent the relapsing component of over 800 patients, approximately double the size of the largest reported international medulloblastoma clinical trials.

**Implications of all the available evidence**

Our study shows the associations between the clinical and molecular characteristics of medulloblastoma at diagnosis, treatment received, and the nature of relapse and subsequent post-relapse disease course. The scale, clinical orientation, and systematic nature of our study support and challenge specific conclusions from previous studies and has uncovered new relationships. Together, these findings provide an evidence base to support translational opportunities aimed at improved disease management and outcomes, through biology-directed disease surveillance, post-relapse prognostication, and risk-stratified selection of second-line treatment strategies.

## Introduction

Over the past decade, advances have been made in the understanding of medulloblastoma biology at diagnosis. Four consensus molecular subgroups—WNT (MB_WNT_), SHH (MB_SHH_), group 3 (MB_Group3_), and group 4 (MB_Group4_)—are now recognised and underpin the 2016 WHO classification of medulloblastoma (genetically defined as WNT-activated; SHH-activated and *TP53*-mutant and SHH-activated and *TP53*-wildtype; and non-WNT and non-SHH [group3 and group4]).^1^ Molecular profiling of a greater number of tumours at diagnosis has led to the discovery of second-generation MB_Group3_ and MB_Group4_ subtypes.^2^, ^3^ Furthermore, molecular features have prognostic significance and define upfront therapy stratification. For example, molecular features identified in international clinical trials (eg, SJMB12 [NCT01878617] and SIOP-PNET5-MB [NCT02066220]) have led to therapy reduction in groups with a good prognosis (eg, MB_WNT_) and treatment intensification or the use of novel therapies for groups at a high risk of relapse and mortality (eg, *MYC-*amplified MB_Group3_).

Disease relapse is the most adverse prognostic factor in medulloblastoma. Relapses occur in approximately 30% of patients and are almost always fatal.^4^, ^5^, ^6^ Most patients will relapse at distant CNS sites with or without disease in the original tumour bed. Individual reports indicate relapse can occur more than 5 years after diagnosis, with the duration of survival after relapse equally as variable.^3^, ^4^, ^5^, ^7^ Durable remissions following relapse typically cluster in younger children (aged <3–5 years) who received deferred craniospinal irradiation as salvage therapy.^8^, ^9^ Older children (aged >3–5 years) with disease relapse who received conventional upfront therapy (neurosurgery, craniospinal irradiation, and chemotherapy) are treated with various strategies at relapse, including metronomic therapy, high-dose chemotherapy, intrathecal medication, and re-irradiation, but these approaches are commonly unsuccessful (typically <5% long-term survival).^6^, ^7^, ^10^, ^11^, ^12^, ^13^ Of note, few studies have attempted to characterise the nature of medulloblastoma relapse, its clinical and molecular correlates, and their translational potential.^5^, ^7^

Here, we report the nature of medulloblastoma relapse (ie, timings, radiological patterns, and post-relapse outcomes), and its associations with treatment and both established and contemporary clinical and molecular features, in a cohort of patients with medulloblastoma who relapsed. This study encompasses all established disease-wide molecular features and second-generation MB_Group3_ and MB_Group4_ subtypes.^2^, ^3^

## Methods

### Study design and participants

In this multicentre cohort study, patients aged 0–18 years at diagnosis with medulloblastoma who had relapsed following upfront therapy were assembled from 16 UK Children's Cancer and Leukaemia Group institutions and four collaborating centres. Eligible patients had a confirmed diagnosis of medulloblastoma, a defined period of remission following upfront therapy, and a documented relapse on imaging or biopsy. According to current treatment conventions, infants (aged <3–5 years, cutoff depending on national treatment protocols) commonly received radiotherapy-sparing treatment at diagnosis; whereas children (aged >3–5 years) received conventional multimodal therapy, which included craniospinal irradiation.^14^ Therefore, we distinguished two restricted subcohorts for specific analyses: patients older than 3 years who received craniospinal irradiation at diagnosis (irradiated group) and patients younger than 5 years who did not receive craniospinal irradiation (non-irradiated group).

Pathological variant was assigned according to 2016 WHO criteria.^1^ Metastatic status at diagnosis was determined according to Chang's criteria.^15^ All patients in the restricted irradiated group received conventional radiotherapy protocols (mode 36/54 Gy; range 24–40/50–60 Gy) following maximal safe neurosurgical resection. In the non-irradiated group, some patients received focal radiotherapy (mode 54 Gy; range 45–55 Gy). Institutional imaging reports were collated and centrally reviewed by an experienced panel of paediatric neuro-oncologists (RMH and SB) to assess the degree of surgical resection (subtotal resection or gross total resection) and patterns of relapse. When interpretation of reported patterns was unclear, clarification was sought with the originating centre or clinical team. Patients with nodular relapses (defined as single or multiple discrete lesions) were distinguished from patients with diffuse (ie, diffuse leptomeningeal dissemination) or mixed-pattern relapses. Similarly, patients with local relapses were considered separately from patients with either distant or combined local and distant relapses. All patients had a documented clinical remission on imaging before relapse.^16^

### Procedures

Established disease-wide molecular features either accepted in the WHO classification of medulloblastoma, or used as the current basis for treatment stratification, were evaluated.^1^ These comprised molecular subgroup (MB_WNT_, MB_SHH_, MB_Group3_, and MB_Group4_), *TP53* mutation status, and *MYC* or *MYCN* oncogene amplification.^17^ Additionally, second-generation methylation subtypes, isochromosome 17q, and *TERT* mutations were evaluated as additional features with potential clinical relevance.^2^, ^3^, ^18^, ^19^, ^20^

Tumours were assigned to consensus molecular subgroups, as previously described using non-negative matrix factorisation and t-distributed stochastic neighbor embedding with density-based spatial clustering of applications with noise clustering.^21^ For samples with a low yield of DNA, the minimal methylation classifier was used.^22^ For MB_Group3_ and MB_Group4_ in the irradiated group, second-generation high-risk and low-risk subgroups were assigned as previously described,^2^ and subtypes I–VIII were identified according to the Group 3 and Group 4 classifier.^3^ *TP53* mutation status was assessed for exons 4–9 using established methods.^4^ Copy-number assessment by interphase fluorescent in-situ hybridisation of *MYC* (8q24·21 probes), *MYCN* (2p24·3 probes), and chromosome 17 imbalances (17p13·3 and 17q12 probes) was done as previously described.^4^ *TERT* promoter mutation detection (mutations 228C→T and 250C→T) was done according to previous reports.^19^ An additional locked nucleic acid probe was designed to detect the 228C→A mutation (*TERT* mut 228C→A, 5′-56FAM-CCC CAT CCG G-3IABkFQ-3′; Integrated DNA Technologies, Coralville, IO, USA). Genotyping for this mutation was done separately with a probe targeted to the wild type sequence (*TERT* WT, 5′-5HEX-CCC CTC CCG G-3IABkFQ-3′; Integrated DNA Technologies, Coralville, IO, USA) using the same method.

### Statistical analysis

Individual missing datapoints were missing completely at random. All recurrently detected (ie, occurred twice or more) established features were tested. Second-generation MB_Group3_ and MB_Group4_ methylation subtypes which were present in more than 10% of patients with MB_Group3_ and MB_Group4_ tumours treated with craniospinal irradiation, were also included for subanalyses. χ^2^ and Fisher's exact tests were used to assess associations between clinical and molecular features. The log-rank test was used in univariable analyses to assess time to relapse, time from relapse to death, and overall survival, and the Kaplan-Meier method was used to visualise results. Cox proportional-hazards models were used to investigate the significance of all covariates for time to relapse, time to death after relapse, and overall survival in univariable and multivariable models, using forward likelihood-ratio testing. In the time to death and overall survival analyses, data were censored for patients who died of other causes or were alive with disease. We tested the proportionality assumption for Cox modelling using scaled Schoenfeld residuals. Proportional covariates with a raw p value of less than 0·1 in univariable analyses were taken forward as candidates for multivariable modelling. Multivariable models were restricted to three covariates in the entire cohort, and two covariates for all subcohorts. The Benjamini-Hochberg procedure was used to control the false discovery rate and adjusted p values of less than 0·05 identified significant associations. Analysis and visualisation was done using GraphPad Prism (version 8.4.1) and the R statistical environment (version 3.5.3).

### Role of the funding source

The funders had no role in study design, cohort collection, analyses, data interpretation, or writing of the report. RMH, ECS, GR, DW, and SCC had access to raw data. The corresponding author had full access to all data and had final responsibility for the decision to submit for publication.

## Results

247 patients (175 [71%] boys and 72 [29%] girls) with medulloblastoma relapse were included in the study (table 1). 17 patients were later excluded from analyses because they did not meet the age and treatment criteria for inclusion. 34 (15%) patients had tumour samples collected in 2010–14, 87 (37%) in 2000–09, 90 (39%) in 1990–99, and the remaining 21 (9%) with data available were collected before 1990. Year of diagnosis was unavailable in 15 samples. All variables were assessed by era (before 2000 *vs* 2000 and after**).** There were no variables that biased planned analyses (appendix pp 2–4). As expected, age and upfront treatment were confounded (p<0·0001; appendix p 5). Pathological variant was centrally assessed in 222 (90%) patients, local assessment was done for the remaining 25 (10%) participants (table 1).

**Table 1:** Patterns of relapse and clinical and molecular features of the entire cohort and the two age-restricted subcohorts

	**Entire cohort (n=247)**	**Irradiated group (n=178)**	**Non-irradiated group (n=52)**
**Patterns of relapse**			
Nodular	86/187 (46%)	53/125 (42%)	23/47 (49%)
Diffuse	66/187 (35%)	46/125 (37%)	17/47 (36%)
Nodular and diffuse	35/187 (19%)	26/125 (21%)	7/47 (15%)
Local relapse	43/193 (22%)	21/129 (16%)	16/48 (33%)
Distant relapse	96/193 (50%)	73/129 (57%)	16/48 (33%)
Combined relapse	54/193 (28%)	35/129 (27%)	16/48 (33%)
**Clinical features at disease relapse**			
Boys	175/247 (71%)	133/178 (75%)	32/52 (62%)
Girls	72/247 (29%)	45/178 (25%)	20/52 (38%)
Boys:girls ratio	2·4:1	3:1	1·6:1
Infants (<4·0 years)	38/247 (15%)	1/178 (1%)	32/52 (62%)
Children (4·0–16·0 years)	176/247 (72%)	145/178 (81%)	20/52 (38%)
Adults (>16·0 years)	33/247 (13%)	32/178 (18%)	0/52
**Treatment at disease relapse**			
Resection	47/182 (26%)	32/124 (26%)	12/45 (27%)
Chemotherapy	128/185 (69%)	102/127 (80%)	22/46 (48%)
Craniospinal irradiation	19/188 (10%)	1/127 (1%)	15/48 (31%)
Focal radiotherapy	23/188 (12%)	18/127 (14%)	4/48 (8%)
**Clinicopathological features and treatment at diagnosis**			
Infants (<4·0 years)	67/247 (27%)	11/178 (6%)	48/52 (92%)
Children (4·0–16·0 years)	172/247 (70%)	160/178 (90%)	4/52 (8%)
Adults (>16·0 years)	8/247 (3%)	7/178 (4%)	0/52
Subtotal resection	78/245 (32%)	58/178 (33%)	14/52 (27%)
Chemotherapy	206/243 (84%)	142/177 (80%)	49/50 (98%)
Craniospinal irradiation	187/247 (76%)	178/178 (100%)	0/178
Classic histology	158/226 (70%)	123/162 (76%)	28/48 (58%)
Large-cell anaplastic histology	39/226 (17%)	26/162 (16%)	8/48 (17%)
Desmoplastic/nodular histology	29/226 (13%)	13/162 (8%)	12/48 (25%)
Distant disease	77/238 (32%)	57/170 (34%)	13/52 (25%)
**Established molecular features at diagnosis**			
*MYC* amplification	13/220 (6%)	5/157 (3%)	6/48 (13%)
*MYCN* amplification	24/217 (11%)	20/155 (13%)	2/47 (4%)
*TP53* mutation	19/216 (9%)	18/150 (12%)	0/49
Isochromosome 17q	50/167 (30%)	40/117 (34%)	6/38 (16%)
*TERT* mutation	14/221 (6%)	10/162 (6%)	2/44 (5%)
MB_WNT_	5/216 (2%)	5/156 (3%)	0/44
MB_SHH_	60/216 (28%)	29/156 (19%)	25/44 (57%)
MB_Group3_	62/216 (29%)	40/156 (26%)	15/44 (34%)
MB_Group4_	89/216 (41%)	82/156 (53%)	4/44 (9%)

Data are n/N (%). MB=medulloblastoma. 17 patients from the entire cohort were excluded because they did not meet the restricted cohort criteria.

**Figure 1:**
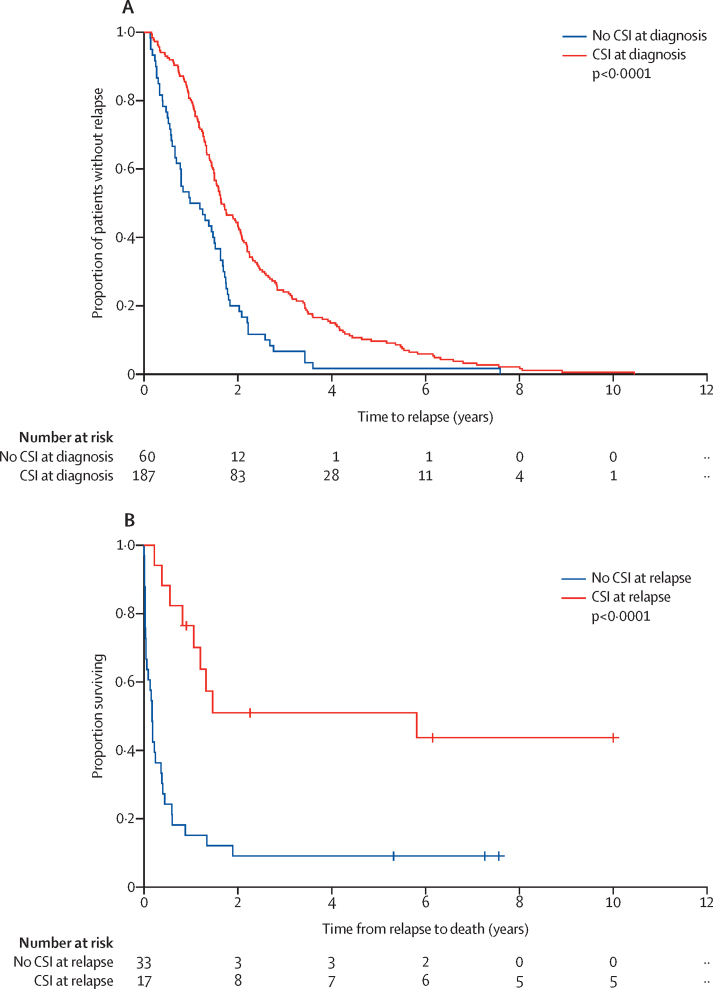
Time to relapse and survival after medulloblastoma relapse according to treatment received (A) Time to relapse according to whether CSI was delivered at diagnosis. (B) Time from relapse to death or last follow-up in patients who did not receive upfront CSI, according to whether CSI was delivered at relapse. Ten patients were removed from time from relapse to death analysis due to: death from other cause (n=1), alive with disease (n=5), or missing data (n=4). CSI=craniospinal irradiation.

We first characterised relapse-specific features—including patterns of relapse (eg, nodular or diffuse disease) and treatment history—and established disease features at diagnosis in the entire cohort (table 1) and assessed these against time to relapse and overall survival. In univariable analyses of the entire cohort, upfront craniospinal irradiation significantly prolonged time to relapse (p<0·0001; figure 1A). Similarly, craniospinal irradiation at relapse was associated with an improved overall survival (p=0·0024; table 2). In 60 patients who did not receive upfront craniospinal irradiation, craniospinal irradiation at relapse was associated with survival after relapse (p<0·0001; figure 1B). Multivariable Cox modelling showed that any radiotherapy at diagnosis significantly increased time to relapse; whereas large-cell anaplastic histology and MB_Group3_ tumour subgroup significantly decreased time to relapse. In the entire cohort, nodular relapses and radiotherapy at relapse significantly increased overall survival and *MYC* amplification significantly decreased overall survival (table 2).

**Table 2:** Univariable and multivariable analyses of correlates of time to relapse and overall survival in the entire cohort

	**Frequency**	**Univariable analyses**				**Multivariable analyses**			
		Time to relapse		Overall survival		Time to relapse		Overall survival	
		HR (95% CI)	p value	HR (95% CI)	p value	HR (95% CI)	p value	HR (95% CI)	p value
**Patterns of disease relapse**									
Nodular	86/187 (46%)	..	..	0·70 (0·51–0·97)	0·096	..	..	0·61 (0·42–0·89)	0·010
Distant relapse	150/193 (78%)	..	..	1·09 (0·74–1·62)	0·78	..	..	..	..
**Treatment at disease relapse**									
Resection	47/182 (26%)	..	..	0·50 (0·34–0·74)	0·0026	..	..	..	..
Chemotherapy	128/185 (69%)	..	..	0·70 (0·50–0·99)	0·12	..	..	..	..
Craniospinal irradiation	19/188 (10%)	..	..	0·29 (0·15–0·58)	0·0024	..	..	..	..
Focal radiotherapy	23/188 (12%)	..	..	0·64 (0·36–1·14)	0·27	..	..	..	..
Any radiotherapy	42/188 (22%)	..	..	0·39 (0·25–0·62)	0·00057	..	..	0·40 (0·24–0·67)	0·00052
**Clinicopathological features and treatment at diagnosis**									
Boys	175/247 (71%)	0·92 (0·70–1·22)	0·68	0·10 (0·74–1·35)	0·98	..	..	..	..
Infants (<4 years)	67/247 (27%)	1·32 (0·99–1·75)	0·15	0·84 (0·61–1·17)	0·52	..	..	..	..
Subtotal resection	78/245 (32%)	1·01 (0·77–1·32)	0·95	1·35 (1·00–1·81)	0·11	..	..	..	..
Chemotherapy	206/243 (85%)	1·15 (0·80–1·64)	0·65	1·08 (0·74–1·58)	0·77	..	..	..	..
Craniospinal irradiation	187/247 (76%)	0·52 (0·39–0·70)	0·00011	0·95 (0·68–1·34)	0·81	..	..	..	..
Focal radiotherapy	18/247 (7%)	1·05 (0·65–1·70)	0·88	1·33 (0·76–2·35)	0·50	..	..	..	..
Any radiotherapy	205/247 (83%)	0·38 (0·27–0·54)	<0·0001	1·06 (0·72–1·58)	0·83	0·34 (0·23–0·50)	<0·0001	..	..
Classic histology	158/226 (70%)	0·69 (0·52–0·92)	0·030	0·89 (0·65–1·22)	0·62	..	..	..	..
Large-cell anaplastic histology	39/226 (17%)	1·64 (1·16–2·33)	0·018	2·17 (1·49–3·17)	0·00083	1·62 (1·12–2·34)	0·0097	..	..
Desmoplastic/nodular histology	29/226 (13%)	1·13 (0·76–1·67)	0·69	0·57 (0·36–0·91)	0·075	..	..	..	..
Distant disease	77/238 (32%)	1·19 (0·90–1·56)	0·44	1·08 (0·80–1·45)	0·76	..	..	..	..
**Established molecular features at diagnosis**									
*MYC* amplification	13/220 (6%)	3·80 (2·15–6·72)	<0·0001	5·39 (3·01–9·62)	<0·0001	..	..	6·18 (3·17–12·05)	<0·0001
*MYCN* amplification	24/217 (11%)	1·27 (0·83–1·94)	0·50	1·69 (1·07–2·68)	0·082	..	..	..	..
*TP53* mutation	19/216 (9%)	1·22 (0·76–1·96)	0·65	1·3 (0·79–2·12)	0·54	..	..	..	..
Isochromosome 17q	50/167 (30%)	0·82 (0·48–1·42)	0·70	1·17 (0·82–1·68)	0·57	..	..	..	..
*TERT* mutation	14/221 (6%)	0·92 (0·66–1·29)	0·66	1·17 (0·68–2·02)	0·73	..	..	..	..
MB_WNT_	5/216 (2%)	0·66 (0·27–1·60)	0·59	0·56 (0·14–2·27)	0·59	..	..	..	..
MB_SHH_	60/216 (28%)	1·32 (0·98–1·78)	0·15	0·82 (0·58–1·16)	0·50	..	..	..	..
MB_Group3_	62/216 (29%)	1·82 (1·35–2·46)	0·00041	1·92 (1·40–2·65)	0·00044	1·83 (1·33–2·54)	0·00024	..	..
MB_Group4_	89/216 (41%)	0·55 (0·41–0·72)	0·00011	0·75 (0·55–1·00)	0·12	..	..	..	..

HR=hazard ratio. MB=medulloblastoma.

Considering these radiotherapy-associated survival differences and the established association between age at diagnosis and upfront craniospinal irradiation,^23^ we divided the cohort into two groups: 178 children were included in the irradiated group and 52 children were included in the non-irradiated group (table 1). All subsequent analyses were done independently within these two restricted groups.

**Figure 2:**
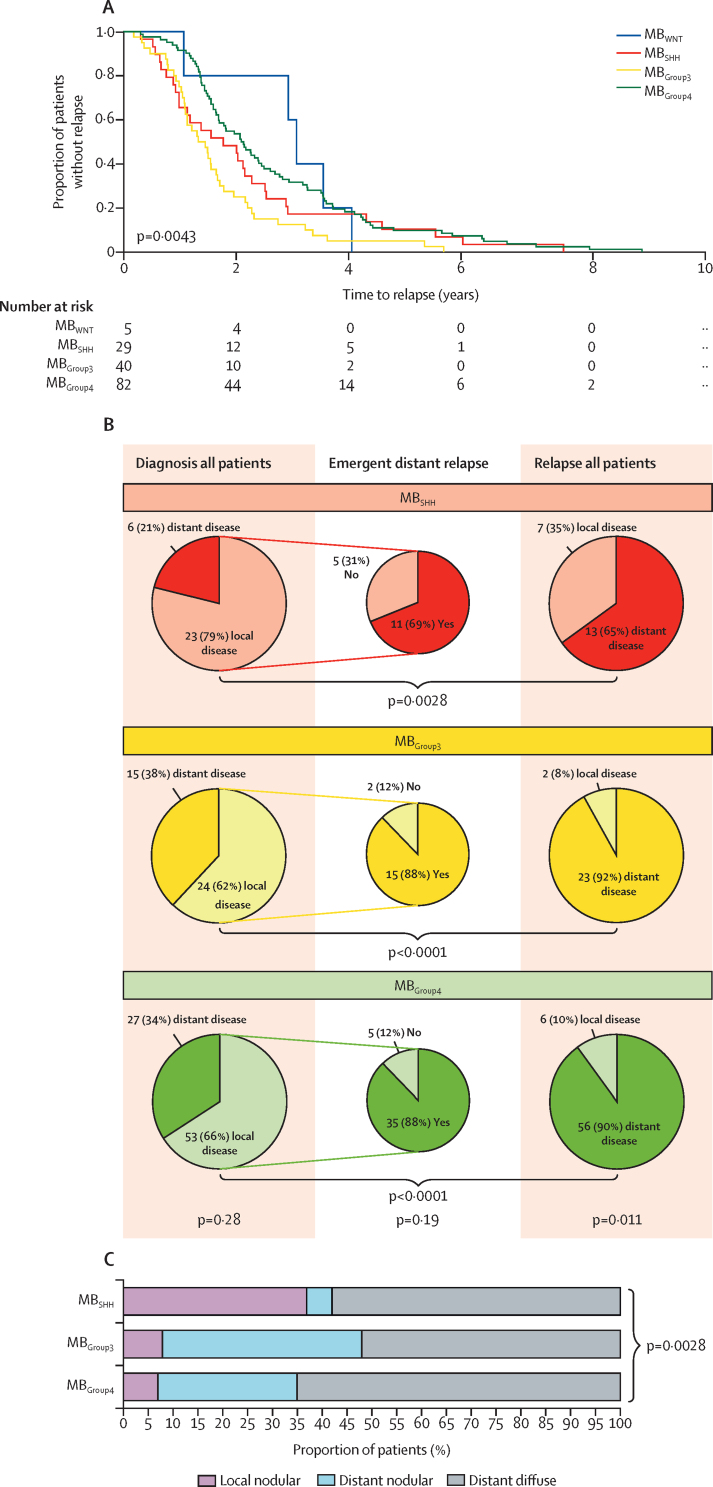
Time to relapse and pattern of relapse according to molecular subgroup in the irradiated group (A) Time to relapse in the four consensus molecular subgroups in patients who received upfront CSI (irradiated group). (B) Schematic representing the patients with distant disease at diagnosis, emergent distant disease at relapse, and distant disease at relapse according to molecular subgroup. (C) Different patterns of relapsed disease according to molecular subgroup and relapse pattern. MB_SHH_=19 patients, MB_Group3_=25 patients, MB_Group4_=58 patients. CSI=craniospinal irradiation. MB=medulloblastoma.

In univariable survival analyses of the irradiated group across all time-based measures, patients with MB_Group3_ tumours had a shorter time to relapse (median 1·34 years [IQR 0·99–1·89]) than did those with MB_Group4_ tumours (2·04 years [1·39–3·42]; p=0·0043; figure 2A). Overall, 18 (10%) of 178 patients in the irradiated group relapsed after 5 years surveillance; of 13 patients with available molecular subgroup data, eight (62%) had MB_Group4_ tumours. Of note, no other feature examined in univariable analyses, including molecular features, such as *MYCN* amplification and *TP53* mutation at diagnosis, were associated with a more rapid disease course (table 3). In multivariable Cox modelling, MB_Group3_ and *MYC* amplification were independent adverse prognostic factors for time to relapse (table 3). Large-cell anaplastic histology and *MYC* amplification were independent adverse risk factors for overall survival. Large-cell anaplastic histology was associated with reduced time to death from relapse (appendix p 6). Of note, molecular subgroup was not associated with time to death from relapse (appendix p 6).

**Table 3:** Univariable and multivariable analyses of correlates of time to relapse in the irradiated group

	**Frequency**	**Univariable analyses**		**Multivariable analyses**	
		HR (95% CI)	p value	HR (95% CI)	p value
**Clinicopathological features and treatment at diagnosis**					
Boys	133/178 (75%)	0·98 (0·69–1·38)	0·90	..	..
Subtotal resection	58/178 (33%)	0·92 (0·67–1·27)	0·84	..	..
Chemotherapy	142/177 (80%)	1·06 (0·72–1·54)	0·83	..	..
Classic histology	123/162 (76%)	0·83 (0·58–1·19)	0·71	..	..
Large-cell anaplastic histology	26/162 (16%)	1·42 (0·93–2·18)	0·43	..	..
Desmoplastic/nodular histology	13/162 (8%)	0·89 (0·50–1·58)	0·79	..	..
Distant disease	57/170 (34%)	1·29 (0·93–1·78)	0·39	..	..
**Established molecular features at diagnosis**					
*MYC* amplification	5/157 (3%)	3·13 (1·27–7·68)	0·069	16·77 (5·37–52·45)	<0·0001
*MYCN* amplification	20/155 (13%)	1·28 (0·80–2·05)	0·63	..	..
*TP53* mutation	18/150 (12%)	1·40 (0·85–2·31)	0·51	..	..
Isochromosome 17q	40/117 (34%)	0·87 (0·59–1·28)	0·78	..	..
*TERT* mutation	10/162 (6%)	0·76 (0·40–1·46)	0·73	..	..
MB_WNT_	5/156 (3%)	0·75 (0·31–1·84)	0·77	..	..
MB_SHH_	29/156 (19%)	1·10 (0·73–1·65)	0·79	..	..
MB_Group3_	40/156 (26%)	1·88 (1·30–2·72)	0·012	1·77 (1·21–2·59)	0·0031
MB_Group4_	82/156 (53%)	0·65 (0·47–0·89)	0·063	..	..

HR=hazard ratio. MB=medulloblastoma.

In univariable survival analyses of the non-irradiated group, high-risk features (isochromosome 17q, large-cell anaplastic histology, and *MYC* amplification) were associated with a more rapid time to relapse and subsequent poorer overall survival (appendix p 7). In univariable analyses, desmoplastic/nodular histology, MB_SHH_ tumours, and craniospinal irradiation at relapse were associated with a sustained overall survival (appendix p 7). In multivariable Cox modelling, *MYC* amplification and large-cell anaplastic histology (appendix p 7) were significant independent risk factors for reduced time to relapse whereas desmoplastic/nodular histology (hazard ratio [HR] 0·23, 95% CI 0·07–0·77) and craniospinal irradiation at relapse were significantly associated with increased time to death after relapse (0·27, 0·11–0·68; appendix p 7). *MYC* amplification was a significant independent risk factor for decreased overall survival (23·52, 4·85–114·05); however, resection at relapse was associated with longer overall survival (0·17, 0·05–0·57; appendix p 7).

Nine (5%) of 178 patients in the irradiated group survived their relapse. Median follow-up after relapse was 4·72 years (IQR 3·54–10·15), with no common factors observed in these survivors (appendix p 8). 11 (21%) of 52 patients in the non-irradiated group survived their relapse, with a median follow-up after relapse of 7·56 years (6·15–15·40 appendix p 8). Consistent with univariable survival analyses (appendix p 7), desmoplastic/nodular histology was the most frequently observed histological subtype in survivors in this group (seven [64%] of 11 patients); most survivors received craniospinal irradiation (eight [73%] of 11 patients) and had re-resection (six [60%] of ten patients with available data) at relapse. The seven survivors with tumours with desmoplastic/nodular histology were MB_SHH_ (MB_SHH-DN_; appendix p 8). Overall, seven [64%] of 11 patients with MB_SHH-DN_ tumours survived their relapse. Of these seven survivors with MB_SHH-DN_ tumours, all received radiotherapy at relapse (five received craniospinal irradiation and two received focal irradiation; appendix p 8). Of note, of the four patients with MB_SHH-DN_ who died, two had data on their treatment history available; both patients did not receive any radiotherapy at relapse.

Patterns of disease relapse were also molecular subgroup dependent in the irradiated group. Isolated local relapses were most commonly observed in patients with MB_SHH_ tumours (seven [35%] of 20 relapses; p=0·0028); whereas, patients with MB_Group3_ and MB_Group4_ tumours had predominantly distant relapses (figure 2B). Although distant relapses were also predominant in patients with MB_SHH_ tumours (13 [65%] of 20 relapses), these were at a significantly lower frequency than in patients with MB_Group3_ tumours (23 [92%] of 25) and MB_Group4_ tumours (56 [90%] of 62; p=0·011; figure 2B). However, rates of emergence of distant disease at relapse in the irradiated group did not differ between molecular subgroups (p=0·19; figure 2B). Approximately 80% of all patients with local-only disease at diagnosis displayed emergent distant disease at relapse (figure 2B).

Of the 20 isolated local relapses, 19 were significantly associated with nodular disease (p<0·0001; appendix p 5). However, although patients who locally relapsed with MB_SHH_ tumours were significantly associated with nodular disease (p=0·016; appendix p 5) this finding was not true for patients who locally relapsed with MB_Group3_ and MB_Group4_ tumours, because of their differing patterns of distant relapse. Patients with MB_SHH_, MB_Group3_, and MB_Group4_ tumours all had distant-diffuse relapses (figure 2C). However, distant nodular relapses were almost exclusively observed in patients with MB_Group3_ and MB_Group4_ tumours (figure 2C). Furthermore, in post-hoc exploratory analyses, nine (82%) of 11 patients with distantly relapsed diffuse MB_SHH_ tumours had *MYCN* amplification at diagnosis, compared with two [25%] of eight patients with nodular MB_SHH_ relapses (p=0·024; appendix p 5). Distant diffuse patterns were associated with early relapses (within 18 months of initial diagnosis) in patients in the irradiated group with MB_Group3_ tumours (p=0·0048; appendix p 5), this association was not observed in patients with MB_SHH_ or MB_Group4_ tumours.

Subgroup-specific patterns of relapse were also observed in the non-irradiated group. Patients with MB_SHH_ (p<0·0001) and MB_Group3_ (p=0·060) tumours acquired distant relapses (appendix p 9) and had predominantly distant disease at relapse (17 [71%] of 24 MB_SHH_ tumours and 12 [80%] of 15 MB_Group3_ tumours; appendix p 9). MB_SHH_ and MB_Group3_ displayed similar proportions of distant-diffuse and distant-nodular relapses (p=0·71; appendix p 9); local-nodular relapses were more commonly observed in patients with MB_SHH_ tumours (appendix p 9).

**Figure 3:**
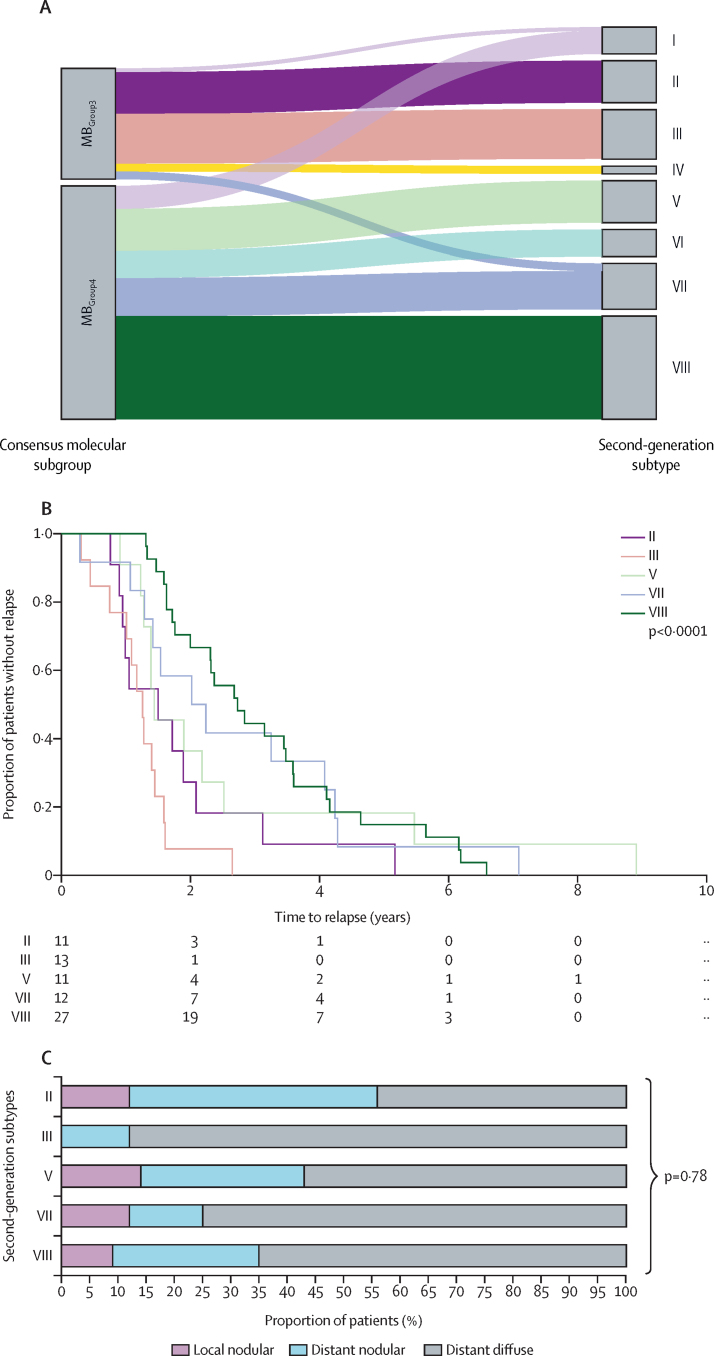
Assessment of relapse characteristics by second-generation MB_Group3_ and MB_Group4_ subtypes (A) Sankey plot of the relationship between consensus MB_Group3_ and MB_Group4_ and second-generation subtypes I–VIII. (B) Time to relapse according to second-generation MB_Group3_ and MB_Group4_ molecular subtype. (C) The different patterns of relapsed disease according to second-generation MB_Group3_ and MB_Group4_ molecular subtype. Subtype II=9 patients. Subtype III=8 patients. Subtype V=7 patients. Subtype VII=8 patients. Subtype VIII=22 patients. MB=medulloblastoma.

Exploratory analyses of second-generation molecular subtypes within MB_Group3_ and MB_Group4_ in the irradiated group showed further insights into the patterns and timings of relapse. Analyses of MB_Group3_ and MB_Group4_ subtypes I–VIII (figure 3A) in 90 patients revealed a differing disease course between subtypes (time to relapse p<0·0001; time from relapse to death p=0·00012; and overall survival p=0·0086; figure 3B; appendix p 10). For example, group II, associated with MB_Group3_ and *MYC* amplification, showed the shortest time to death after relapse (median 0·26 years [0·09–0·55], p=0·0063; appendix p 11). Similar to the consensus MB_Group3_ and MB_Group4_ molecular subgroups, subtypes II (p=0·0098), III (p=0·0046), and VIII (p<0·0001) developed distant disease at relapse whereas subtypes V (p=0·32) and VII (p=0·16) maintained their distant disease status between diagnosis and relapse (table 4). However, patterns of distant disease at relapse did not differ significantly between subtypes (figure 3C).

**Table 4:** The prevalence of distant disease at diagnosis and distant disease acquired at relapse according to second-generation MB_Group3_ and MB_Group4_ molecular subtype

	Distant disease at diagnosis*	**Distant disease at relapse**†	**p value**‡	**Acquired distant disease**§
II	3/11 (27%)	8/9 (89%)	0·0098	6/7 (86%)
III	4/13 (31%)	8/8 (100%)	0·0046	7/7 (100%)
V	6/11 (55%)	6/7 (86%)	0·32	3/4 (75%)
VII	6/12 (50%)	7/8 (87%)	0·16	2/3 (67%)
VIII	6/27 (22%)	22/24 (92%)	<0·0001	17/19 (89%)

Only MB_Group3_ and MB_Group4_ subtypes present in >10% of cohort included in analyses (I, IV, and VI excluded from analyses). MB=medulloblastoma.

* No significant difference by molecular subtype p=0·25.

† No significant difference by molecular subtype p=0·88.

‡ p value comparing the proportion of distant disease at diagnosis versus distant disease at relapse within MB_Group3_ and MB_Group4_ second-generation subtypes.

§ No significant difference by molecular subtype p=0·58.

Evaluating the methylation subtypes described by Schwalbe and colleagues,^2^ MB_Group3-HR_ had a rapid disease course (time to relapse p<0·0001; time from relapse to death p=0·0011; overall survival p<0·0001; appendix p 12). Rates of emergent distant disease at relapse were similar across subtypes (appendix p 12). In multivariable analyses of the irradiated MB_Group3_ and MB_Group4_ tumours, MB_Group4-HR_ had increased time to relapse whereas subtype III had decreased time to relapse (appendix p 11). Subtype II was associated with a rapid disease course after relapse (appendix p 11), whereas nodular disease at relapse was associated with a prolonged time from relapse to death (HR 0·42, 0·21–0·81). Finally, MB_Group3-HR_ and *MYC* amplification were associated adversely with overall survival (appendix p 11).

## Discussion

We have presented the largest study of the nature and correlates of disease relapse in patients with medulloblastoma. Our results suggest that treatment and molecular subgroup are associated with the nature of relapse and subsequent disease course. These findings can help to inform disease management through improved post-therapy surveillance and post-relapse prognostication.

In the irradiated group, disease course varies according to molecular subgroup; patients with MB_Group4_ tumours were associated with a prolonged time to relapse (appendix p 14) and consequently warrant a prolonged surveillance period of up to 10 years. Furthermore, patterns of relapse were molecular subgroup dependent. Local-nodular relapses were more common in MB_SHH_ tumours as previously reported.^5^ However, MB_SHH_, MB_Group3_, and MB_Group4_ molecular subgroups all developed a significant percentage of distant relapses. Of note, this finding does not support suggestions by others to intensify the local radiotherapy boost delivered to patients with MB_SHH_ tumours at diagnosis to reduce relapse rates; isolated local MB_SHH_ relapses in our cohort were in the minority.^5^

Our study details the subgroup specific patterns of distant relapse that can aid both disease surveillance and post-relapse prognostication (appendix p 14). Following craniospinal irradiation, distantly relapsed MB_SHH_ almost exclusively displayed diffuse disease, which is associated with *MYCN* amplification at diagnosis. Therefore, MRI surveillance after initial treatment of patients with *MYCN* amplified MB_SHH_ should always consider the whole neuroaxis, even if the patient had local-only disease at diagnosis. Distantly relapsed MB_Group3_ and MB_Group4_ tumours displayed both nodular and diffuse patterns of disease. Nodular relapse was associated with prolonged survival post-relapse in patients with MB_Group3_ and MB_Group4_ tumours. Together, these findings should be used to inform clinical discussions and treatment decisions. For example, nodular disease is more amenable to local therapy strategies (re-resection and focal irradiation), which might be justified given this new evidence of their prolonged survival after relapse.

Patients not treated with upfront craniospinal irradiation relapsed quickly, with more than half of all children relapsing within 18 months of initial diagnosis. This rapid disease course is compounded by the presence of recognised high-risk features (eg, *MYC* amplification and large-cell anaplastic histology) that inform current clinical management. However, patterns of distantly-relapsed non-irradiated MB_SHH_ and MB_Group3_ tumours differed to those observed in the irradiated group and were not associated with disease course after relapse. Treatment at relapse (eg, craniospinal irradiation at relapse and re-resection), MB_SHH_, and desmoplastic/nodular histology were associated with a more favourable disease course after relapse. Together, these findings inform counselling at diagnosis and relapse, identifying a subcohort of patients with potentially salvageable disease should it relapse (appendix p 14).

Finally, analyses of novel second-generation molecular subtypes suggest specific subtypes further resolve the heterogeneous nature of MB_Group3_ and MB_Group4_ relapses after treatment with craniospinal irradiation. For example subtype II tumours displayed a rapid time to death after relapse. These findings emphasise the importance of understanding tumour biology at diagnosis, how this relates to the nature of disease relapse and underlying mechanisms, and, in the future, how these could influence prognostication after relapse.

However, this study does have some limitations. Although the study is retrospective and is not based on a uniformly treated clinical trial cohort, all patients were managed using current contemporary treatment strategies appropriate to their age. Of note, we considered the retrospective nature of this cohort in our assessments; no variable or outcome measure was biased by treatment era (before 2000 *vs* 2000 and after). To allow us to answer the study questions, only patients who relapsed with disease were selected for this study. Overall, we estimate our cohort comprises approximately 50% of all patients (aged up to 18 years) in the UK with medulloblastoma relapse during the study collection period. By contrast to all previous studies,^5^, ^7^ we have considered upfront-treatment differences, and analysed the patterns and timings of relapse in conjunction with detailed annotation of all established disease-wide features at diagnosis and novel second-generation methylation subtypes.

In summary, consideration of upfront diagnostic disease features, coupled with time and pattern of relapse, offers opportunities to improve the clinical management of medulloblastoma.
