# Supplementary material for: Time, pattern, and outcome of medulloblastoma relapse and their association with tumour biology at diagnosis and therapy: a multicentre cohort study
Source: Lancet Child Adolesc Health. 2020 Dec;4(12):865–74. doi: 10.1016/S2352-4642(20)30246-7 (PMC7671998; doi:10.1016/S2352-4642(20)30246-7)
Supplement: Supplementary appendix [file mmc1.pdf]

THE LANCET
Child & Adolescent Health

Supplementary appendix

This appendix formed part of the original submission. We post it as supplied by the authors.

Supplement to: Hill RM, Richardson S, Schwalbe EC. Time, pattern, and outcome of medulloblastoma relapse and their association with tumour biology at diagnosis and therapy: a multicentre cohort study. *Lancet Child Adolesc Health* 2020; published online October 22, 2020. http://dx.doi.org/10.1016/S2352-4642(20)30246-7.

Appendix

Time, pattern, and outcome of medulloblastoma relapse and their association with tumour biology at diagnosis and therapy: a multicentre cohort study

Rebecca M Hill, Stacey Richardson, Edward C Schwalbe, Debbie Hicks, Janet C Lindsey, Stephen Crosier, Gholamreza Rafiee, Yura Grabovska, Stephen B Wharton, Thomas S Jacques, Antony Michalski, Abhijit Joshi, Barry Pizer, Daniel Williamson, Simon Bailey, Steven C Clifford

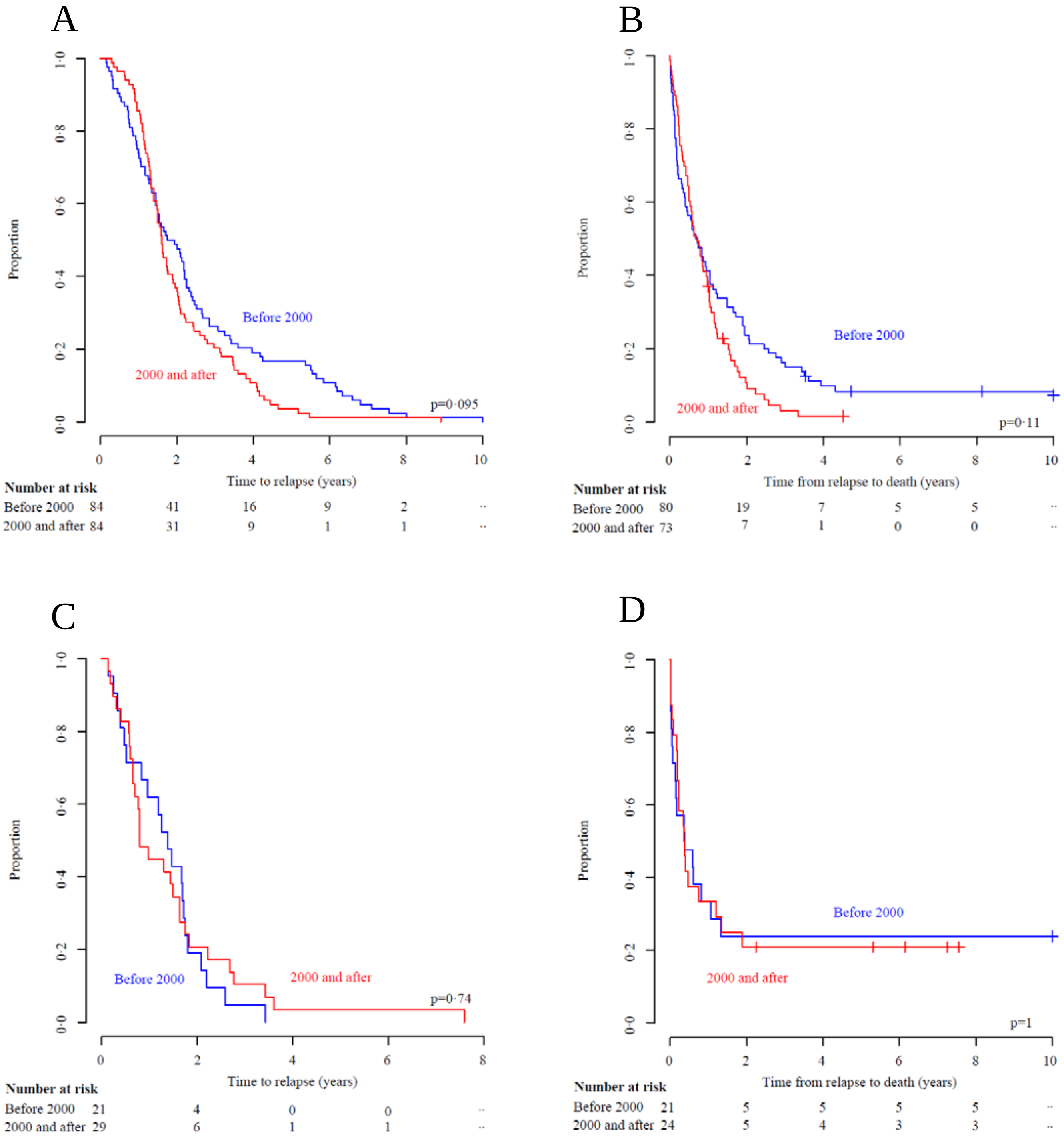

Supplementary Figure 1: Time to relapse and time from relapse to death according to era (before 2000 vs 2000 and after). (A) Time to relapse according to treatment era in the age restricted (>3 years) irradiated group. (B) Time from relapse to death according to treatment era in the age restricted irradiated group. (C) Time to relapse according to treatment era in the age restricted (<5 years) non-irradiated group. (D) Time from relapse to death according to treatment era in the age restricted non-irradiated group.

	Before 2000 (n=84)	2000 and after (n=84)	p value
**Patterns of relapse**			
Nodular	19/41 (46%)	31/76 (41%)	0·70
Distant relapse	35/42 (83%)	64/77 (83%)	1
**Treatment at disease relapse**			
Resection	10/42 (24%)	19/76 (25%)	1
Chemotherapy	28/40 (70%)	66/79 (84%)	0·10
Craniospinal irradiation	NA	NA	NA
Focal radiotherapy	4/43 (9%)	12/76 (16%)	0·41
**Clinicopathological features and treatment at diagnosis**			
Boys	64/84 (76%)	61/84 (73%)	0·72
Subtotal resection*	40/83 (48%)	16/83 (19%)	0·00010
Chemotherapy*	49/84 (58%)	83/83 (100%)	<0·0001
Craniospinal irradiation	All	All	NA
Classic histology	63/80 (78%)	51/72 (71%)	
Large-cell anaplastic histology	10/80 (13%)	15/72 (21%)	] 0·38
Desmoplastic/nodular histology	7/80 (9%)	6/72 (8%)	
Distant disease	22/76 (29%)	32/84 (38%)	0·24
**Established molecular features at diagnosis**			
MYC amplification#	5/71 (7%)	0/78 (0%)	0·023
MYCN amplification	6/70 (9%)	13/77 (17%)	0·15
TP53 mutation	3/63 (5%)	12/79 (15%)	0·056
Isochromosome 17q	16/44 (36%)	21/65 (32%)	0·68
TERT mutation	6/75 (8%)	4/79 (5%)	0·53
MB_{WNT}	0/69 (0%)	3/79 (4%)	
MB_{SHH}	12/69 (17%)	15/79 (19%)	] 0·41
MB_{Group3}	20/69 (29%)	20/79 (25%)	
MB_{Group4}	37/69 (54%)	41/79 (52%)	

Data are n/N (%). MB=medulloblastoma. NA=not applicable. *=Chemotherapy at diagnosis was enriched in patients diagnosed in 2000 and after, subtotal resection was enriched in patients diagnosed before 2000. However, neither variable was associated with time to relapse or time to death after relapse in univariable or multivariable survival analyses. #=*MYC* amplification was enriched in patients diagnosed before 2000. However, testing for *MYC* amplification was not era-specific and was uniformly performed on all suitable samples as part of this study.

Supplementary Table 1: Comparison by era (before 2000 vs 2000 and after) of the patterns of relapse, clinical and molecular features in the irradiated group

	Before 2000 (n=21)	2000 and after (n=29)	p value
**Patterns of relapse**			
Nodular	8/18 (45%)	13/27 (48%)	1
Distant relapse	13/19 (68%)	19/27 (70%)	1
**Treatment at disease relapse**			
Resection	6/19 (32%)	5/24 (21%)	0·49
Chemotherapy*	5/20 (25%)	16/24 (67%)	0·0077
Craniospinal irradiation	9/20 (45%)	5/26 (19%)	0·10
Focal radiotherapy	1/20 (5%)	3/26 (12%)	0·62
**Clinicopathological features and treatment at diagnosis**			
Boys	14/21 (67%)	18/29 (62%)	0·77
Subtotal resection	7/21 (33%)	6/29 (21%)	0·35
Chemotherapy	20/21 (95%)	27/27 (100%)	0·44
Focal radiotherapy*	3/21 (14%)	14/29 (48%)	0·016
Classic histology	11/20 (55%)	16/27 (59%)	
Large-cell anaplastic histology	2/20 (10%)	6/27 (22%)	] 0·32
Desmoplastic/nodular histology	7/20 (35%)	5/27 (19%)	
Distant disease	5/21 (24%)	8/29 (28%)	1
**Established molecular features at diagnosis**			
MYC amplification	2/18 (11%)	4/28 (14%)	1
MYCN amplification	0/17 (0%)	2/28 (7%)	0·52
TP53 mutation	0/19 (0%)	0/28 (0%)	1
Isochromosome 17q	1/14 (7%)	4/23 (17%)	0·63
TERT mutation	1/18 (6%)	1/24 (4%)	1
MB_{SHH}	14/18 (78%)	11/25 (44%)	
MB_{Group3}	4/18 (22%)	11/25 (44%)	] 0·060
MB_{Group4}	0/18 (0%)	3/25 (12%)	

Data are n/N (%). MB=medulloblastoma. *=Chemotherapy at relapse and focal radiotherapy at diagnosis were enriched in patients diagnosed in 2000 and after, but neither variable was associated with time to relapse or time to death after relapse in univariable or multivariable survival analyses.

Supplementary Table 2: Comparison by era (before 2000 vs 2000 and after) of the patterns of relapse, clinical and molecular features in the non-irradiated group

	Infant <4.0 years	Child ≥4.0 years
Craniospinal irradiation at diagnosis	19	168
No craniospinal irradiation at diagnosis	48	12

$p<0.0001$

	Nodular	Diffuse or combined (diffuse & nodular)
Local relapse only	19	1
Distant or combined relapse (local & distant)	34	71

$p<0.0001$

	Nodular	Diffuse or combined (diffuse & nodular)
Local relapse only	7	0
Distant or combined relapse (local & distant)	1	11

$p=0.016$

	Nodular	Diffuse or combined (diffuse & nodular)
MYCN amplified	2	9
MYCN balanced	6	2

$p=0.024$

E

	Nodular	Diffuse or combined (diffuse & nodular)
Early relapse	3	11
Late relapse	9	2

$p=0.0048$

Supplementary Figure 2: Contingency tables illustrating significant associations observed. (A) Age and upfront treatment in the entire cohort. (B) Location and pattern of disease relapse in the irradiated group. (C) Location and pattern of MB_{SHH} relapse in the irradiated group. (D) Diffuse MB_{SHH} tumours frequently display *MYCN* amplification in the irradiated group. (E) Diffuse patterns in MB_{Group3} are associated with early relapses (within 18 months of initial diagnosis) in the irradiated group.

	Frequency	Univariable analyses				Multivariable analyses			
		Time to death after relapse		Overall survival		Time to death after relapse		Overall survival	
		HR (95% CI)	p value	HR (95% CI)	p value	HR (95% CI)	p value	HR (95% CI)	p value
**Patterns of disease relapse**									
Nodular	53/125 (42%)	0·55 (0·37-0·82)	0·064	0·59 (0·40-0·88)	0·063	··	··	··	··
Distant relapse	108/129 (84%)	1·57 (0·92-2·67)	0·29	1·28 (0·75-2·18)	0·55	··	··	··	··
**Treatment at disease relapse**									
Resection	32/124 (26%)	0·72 (0·46-1·13)	0·33	0·63 (0·40-0·98)	0·15	··	··	··	··
Chemotherapy	102/127 (80%)	0·54 (0·34-0·86)	0·095	0·65 (0·41-1·03)	0·19	··	··	··	··
Focal radiotherapy	18/127 (14%)	0·73 (0·40-1·34)	0·46	0·82 (0·45-1·50)	0·65	··	··	··	··
**Clinicopathological features and treatment at diagnosis**									
Boys	133/178 (75%)	1·27 (0·88-1·83)	0·36	1·21 (0·84-1·75)	0·50	··	··	··	··
Subtotal resection	58/178 (33%)	1·31 (0·94-1·84)	0·27	1·15 (0·82-1·62)	0·59	··	··	··	··
Chemotherapy	142/177 (80%)	1·15 (0·77-1·73)	0·61	1·24 (0·82-1·87)	0·53	··	··	··	··
Classic histology	123/162 (76%)	0·77 (0·52-1·14)	0·35	0·74 (0·50-1·09)	0·27	··	··	··	··
Large-cell anaplastic histology	26/162 (16%)	1·66 (1·04-2·65)	0·23	1·70 (1·06-2·71)	0·14	1·66 (1·04-2·65)	0·033	1·82 (1·12-2·97)	0·015
Desmoplastic/nodular histology	13/162 (8%)	0·88 (0·49-1·60)	0·72	0·93 (0·52-1·69)	0·91	··	··	··	··
Distant disease	57/170 (34%)	1·19 (0·85-1·68)	0·44	1·38 (0·98-1·95)	0·17	··	··	··	··
**Established molecular features at diagnosis**									
MYC amplification	5/157 (3%)	2·44 (0·99-6·05)	0·22	3·72 (1·51-9·20)	0·046	··	··	4·21 (1·69-10·50)	0·0020
MYCN amplification	20/155 (13%)	1·58 (0·95-2·64)	0·28	1·51 (0·91-2·52)	0·26	··	··	··	··
TP53 mutation	18/150 (12%)	1·12 (0·67-1·86)	0·75	1·34 (0·80-2·26)	0·51	··	··	··	··
Isochromosome 17q	40/117 (34%)	0·96 (0·64-1·44)	0·86	0·94 (0·63-1·40)	0·88	··	··	··	··
TERT mutation	10/162 (6%)	1·27 (0·66-2·42)	0·62	1·023 (0·54-1·95)	0·99	··	··	··	··
MB_{WNT}	5/156 (3%)	0·44 (0·11-1·81)	0·42	0·60 (0·15-2·42)	0·62	··	··	··	··
MB_{SHH}	29/156 (19%)	1·11 (0·71-1·74)	0·76	1·013 (0·65-1·59)	0·95	··	··	··	··
MB_{Group3}	40/156 (26%)	1·50 (1·02-2·19)	0·20	1·76 (1·20-2·58)	0·080	··	··	··	··
MB_{Group4}	82/156 (52%)	0·75 (0·54-1·06)	0·27	0·69 (0·49-0·98)	0·15	··	··	··	··

HR=hazard ratio. MB=medulloblastoma

Supplementary Table 3: Univariable and multivariable analyses of correlates of time-based measures in the irradiated group

	Frequency	Univariable analyses						Multivariable analyses					
		Time to relapse		Time to death after relapse		Overall survival		Time to relapse		Time to death after relapse		Overall survival	
		HR (95% CI)	p value	HR (95% CI)	p value	HR (95% CI)	p value	HR (95% CI)	p value	HR (95% CI)	p value	HR (95% CI)	p value
**Patterns of disease relapse**													
Nodular	23/47 (49%)	··	··	0·99 (0·49-2·01)	1	0·78 (0·38-1·59)	0·66	··	··	··	··	··	··
Distant relapse	32/48 (67%)	··	··	0·80 (0·38-1·68)	0·73	1·01 (0·48-2·14)	0·98	··	··	··	··	··	··
**Treatment at disease relapse**													
Resection	12/45 (27%)	··	··	0·29 (0·10-0·83)	0·10	0·25 (0·09-0·73)	0·054	··	··	··	··	0·17 (0·05-0·57)	0·0052
Chemotherapy	22/46 (48%)	··	··	0·59 (0·29-1·22)	0·39	0·72 (0·35-1·46)	0·55	··	··	··	··	··	··
Craniospinal irradiation	15/48 (31%)	··	··	0·21 (0·086-0·52)	0·015	0·26 (0·11-0·65)	0·026	··	··	0·27 (0·11-0·68)	0·0054	··	··
Focal radiotherapy	4/48 (8%)	··	··	0·30 (0·04-2·22)	0·40	0·31 (0·04-2·32)	0·47	··	··	··	··	··	··
**Clinicopathological features and treatment at diagnosis**													
Boys	32/52 (62%)	1·05 (0·60-1·87)	1	0·88 (0·45-1·71)	0·82	0·72 (0·37-1·41)	0·56	··	··	··	··	··	··
Subtotal resection	14/52 (27%)	1·71 (0·91-3·20)	0·21	1·56 (0·78-3·11)	0·38	1·87 (0·93-3·74)	0·17	··	··	··	··	··	··
Chemotherapy	49/50 (98%)	··	··	··	··	··	··	··	··	··	··	··	··
Focal radiotherapy	17/52 (33%)	0·47 (0·25-0·88)	0·066	1·27 (0·64-2·55)	0·71	1·07 (0·53-2·16)	0·94	··	··	··	··	··	··
Classic histology	28/48 (58%)	0·58 (0·32-1·07)	0·22	1·59 (0·79-3·18)	0·38	1·17 (0·58-2·34)	0·83	··	··	··	··	··	··
Large-cell anaplastic histology	8/48 (17%)	4·92 (2·05-11·78)	0·0024	2·30 (1·00-5·32)	0·17	5·00 (2·06-12·13)	0·0038	4·26 (1·67-10·83)	0·0024	··	··	··	··
Desmoplastic/nodular histology	12/48 (25%)	0·97 (0·50-1·90)	1	0·28 (0·11-0·73)	0·066	0·31 (0·12-0·81)	0·049	··	··	0·23 (0·07-0·77)	0·018	··	··
Distant disease	13/52 (25%)	1·09 (0·58-2·06)	1	0·63 (0·28-1·38)	0·38	0·71 (0·32-1·57)	0·58	··	··	··	··	··	··
**Established molecular features at diagnosis**													
MYC amplification	6/48 (13%)	7·06 (2·68-18·57)	0·0010	5·18 (1·89-14·25)	0·014	13·14 (4·10-42·06)	0·00028	5·61 (1·96-16·04)	0·0013	··	··	23·52 (4·85-114·05)	<0·0001
MYCN amplification	2/47 (4%)	3·08 (0·69-13·69)	0·28	1·36 (0·32-5·81)	0·85	2·89 (0·65-12·95)	0·33	··	··	··	··	··	··
Isochromosome 17q	6/38 (16%)	3·37 (1·35-8·39)	0·043	1·99 (0·72-5·55)	0·41	3·63 (1·29-10·24)	0·049	··	··	··	··	··	··
TERT mutation	2/44 (5%)	1·58 (0·37-6·68)	0·75	1·02 (0·24-4·28)	0·98	1·32 (0·31-5·58)	0·83	··	··	··	··	··	··
MB$_{SHH}$	25/44 (57%)	0·98 (0·53-1·81)	0·95	0·51 (0·25-1·06)	0·20	0·42 (0·20-0·87)	0·049	··	··	··	··	··	··
MB$_{Group3}$	15/44 (34%)	1·38 (0·73-2·60)	0·51	2·30 (1·10-4·82)	0·11	2·51 (1·20-5·25)	0·057	··	··	··	··	··	··
MB$_{Group4}$	4/44 (9%)	0·48 (0·14-1·57)	0·39	0·77 (0·18-3·26)	0·80	1·10 (0·26-4·66)	0·95	··	··	··	··	··	··

HR=hazard ratio. MB=medulloblastoma

Supplementary Table 4: Univariable and multivariable analyses of correlates of time-based measures in the non-irradiated group

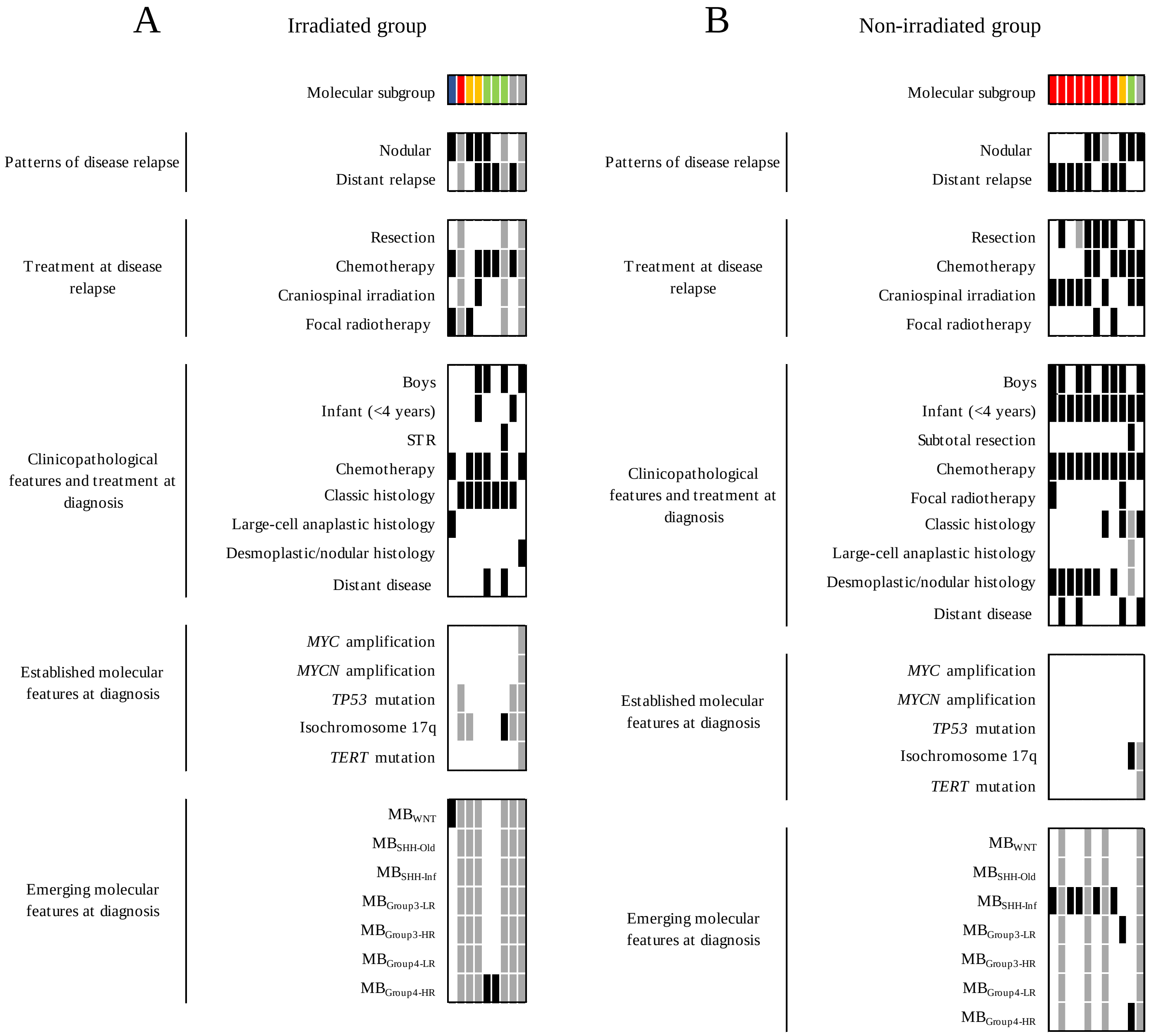

Supplementary Figure 3: Clinical and molecular disease characteristics of all survivors following relapse. (A) Clinical and molecular disease characteristics of survivors in the irradiated group. (B) Clinical and molecular disease characteristics of survivors in the non-irradiated group. Each column represents one patient. Blue box=MB_{WNT}. Red box=MB_{SHH}. Yellow box=MB_{Group3}. Green box=MB_{Group4}. Black box=feature present. Grey box=missing data.

A

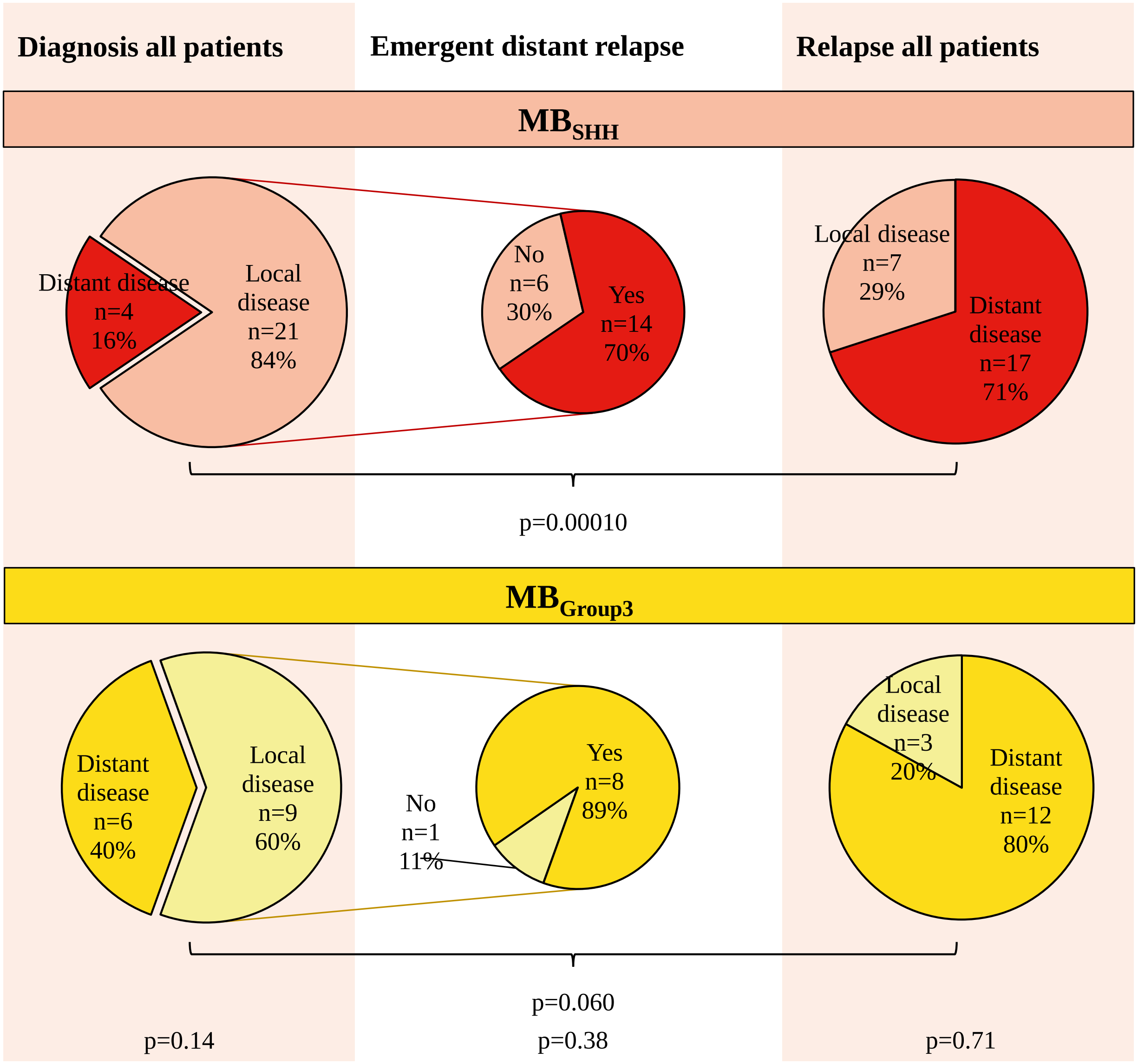

B

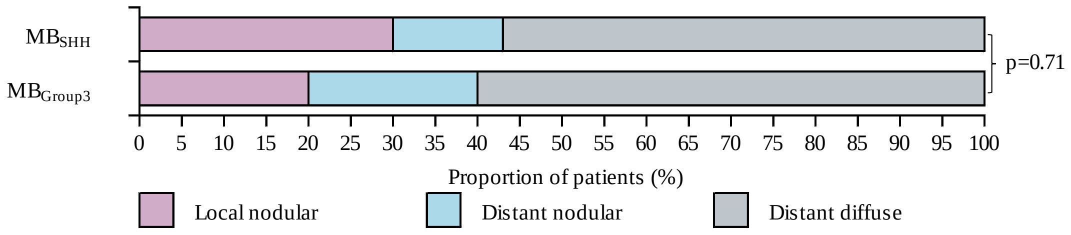

Supplementary Figure 4: Patterns of relapse are molecular subgroup dependent in the non-irradiated group. (A) Schematic representing the patients with distant disease at diagnosis, emergent distant disease at relapse, and distant disease at relapse according to molecular subgroup. (B) Different patterns of relapsed disease according to molecular subgroup and relapse pattern. MB_{SHH}=23 patients, MB_{Group3}=15 patients. MB=medulloblastoma.

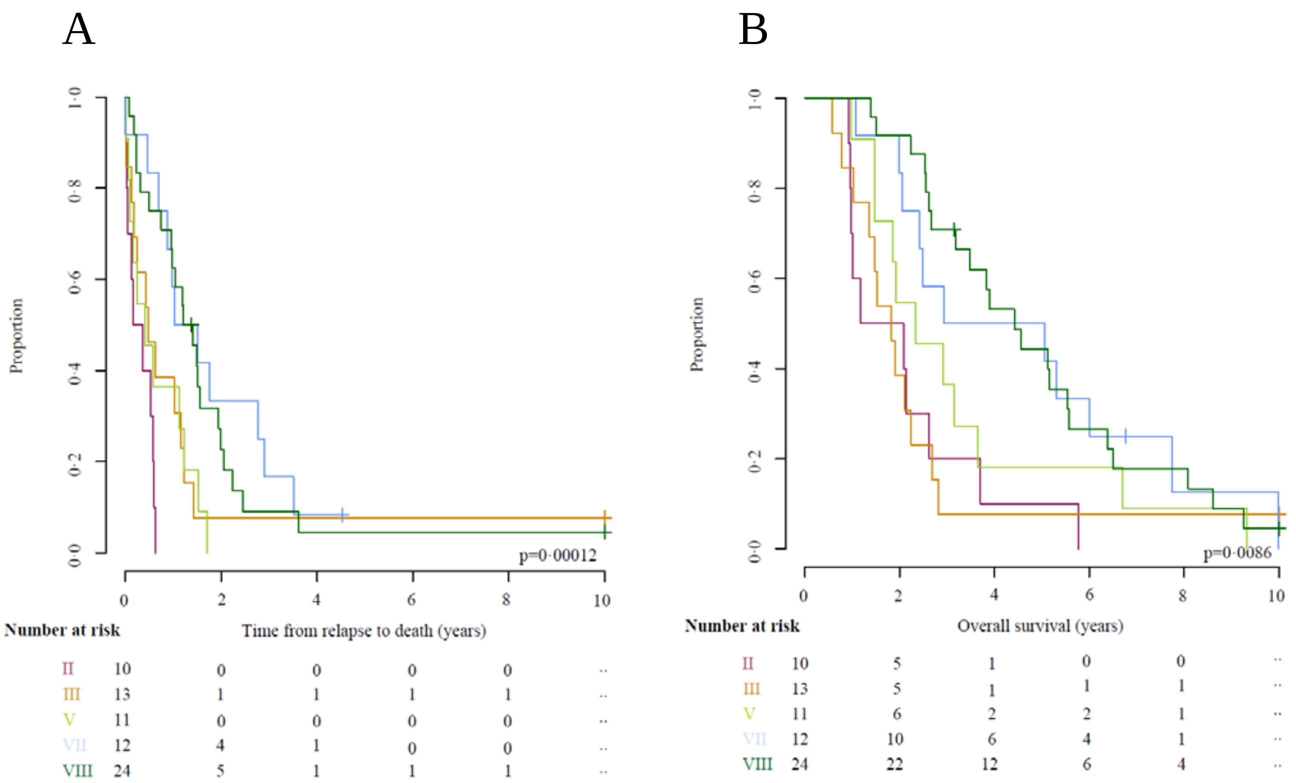

Supplementary Figure 5: Second-generation MB_{Group3} and MB_{Group4} subtypes account for heterogeneous relapse characteristics. (A) Time from relapse to death according to second-generation MB_{Group3} and MB_{Group4} molecular subtype. (B) Overall survival according to second-generation MB_{Group3} and MB_{Group4} molecular subtype. I-VIII=MB_{Group3} and MB_{Group4} second-generation subtypes. Only MB_{Group3} and MB_{Group4} subtypes present in >10% of cohort included in analyses (I, IV and VI excluded from analyses).

	Frequency	Univariable analyses						Multivariable analyses					
		Time to relapse		Time to death after relapse		Overall survival		Time to relapse		Time to death after relapse		Overall survival	
		HR (95% CI)	p value	HR (95% CI)	p value	HR (95% CI)	p value	HR (95% CI)	p value	HR (95% CI)	p value	HR (95% CI)	p value
**Patterns of disease relapse**													
Nodular	32/84 (38%)	··	··	0·50 (0·30-0·81)	0·046	0·51 (0·31-0·83)	0·045	··	··	0·42 (0·21-0·81)	0·010	··	··
Distant relapse	79/87 (91%)	··	··	2·23 (0·96-5·18)	0·18	1·80 (0·784·18)	0·32	··	··	··	··	··	··
**Treatment at disease relapse**								··	··	··	··	··	··
Resection	18/86 (21%)	··	··	0·63 (0·36-1·12)	0·21	0·58 (0·32-1·02)	0·19	··	··	··	··	··	··
Chemotherapy	68/87 (78%)	··	··	0·49 (0·29-0·84)	0·055	0·61 (0·36-1·04)	0·20	··	··	··	··	··	··
Craniospinal irradiation*	1/88 (1%)	··	··	··	··	··	··	··	··	··	··	··	··
Focal radiotherapy	14/88 (16%)	··	··	0·75 (0·38-1·46)	0·58	0·91 (0·47-1·79)	0·94	··	··	··	··	··	··
**Clinicopathological features and treatment at diagnosis**													
Boys	94/122 (77%)	1·05 (0·69-1·61)	0·97	1·15 (0·74-1·78)	0·65	1·19 (0·77-1·85)	0·68	··	··	··	··	··	··
Subtotal resection	41/121 (34%)	0·71 (0·49-1·04)	0·23	1·12 (0·76-1·68)	0·62	0·88 (0·59-1·31)	0·73	··	··	··	··	··	··
Chemotherapy	102/121 (84%)	0·95 (0·58-1·55)	0·93	1·05 (0·63-1·74)	0·90	1·05 (0·63-1·74)	0·93	··	··	··	··	··	··
Classic histology	96/114 (84%)	0·89 (0·53-1·51)	1	0·57 (0·33-0·96)	0·15	0·67 (0·40-1·12)	0·26	··	··	··	··	··	··
Large-cell anaplastic histology	12/114 (11%)	1·28 (0·70-2·33)	0·85	1·91 (1·03-3·53)	0·12	1·66 (0·90-3·07)	0·26	··	··	··	··	··	··
Desmoplastic/nodular histology	6/114 (5%)	0·84 (0·33-2·13)	1	1·33 (0·58-3·06)	0·69	1·17 (0·51-2·68)	0·90	··	··	··	··	··	··
Distant disease	42/119 (35%)	1·52 (1·04-2·24)	0·11	1·15 (0·77-1·70)	0·63	1·38 (0·93-2·06)	0·25	··	··	··	··	··	··
**Established molecular features at diagnosis**													
MYC amplification	3/114 (3%)	19·65 (5·02-76·90)	0·00019	2·90 (0·91-9·28)	0·16	8·45 (2·55-28·04)	0·0061	··	··	··	··	7·33 (1·63-32·80)	0·0092
MYCN amplification	6/114 (5%)	1·16 (0·51-2·65)	0·97	1·51 (0·61-3·73)	0·62	1·57 (0·63-3·88)	0·55	··	··	··	··	··	··
TP53 mutation	2/111 (2%)	0·89 (0·22-3·62)	0·92	0·70 (0·17-2·87)	0·68	0·87 (0·21-3·54)	0·96	··	··	··	··	··	··
Isochromosome 17q	39/89 (44%)	0·84 (0·55-1·29)	0·78	1·03 (0·67-1·60)	0·89	0·97 (0·62-1·50)	0·88	··	··	··	··	··	··
TERT mutation	3/119 (3%)	0·95 (0·30-3·00)	0·92	3·46 (1·06-11·24)	0·14	1·57 (0·50-4·99)	0·65	··	··	··	··	··	··
**Emerging molecular features at diagnosis**													
MB$_{\text{Group3-LR}}$ *	2/78 (3%)	··	··	··	··	··	··	··	··	··	··	··	··
MB$_{\text{Group3-HR}}$	25/78 (32%)	2·68 (1·62-4·43)	0·00061	2·65 (1·55-4·54)	0·0047	3·21 (1·86-5·52)	0·00065	··	··	··	··	3·24 (1·86-5·62)	<0·0001
MB$_{\text{Group4-LR}}$	14/78 (18%)	1·63 (0·89-2·98)	0·25	0·81 (0·44-1·49)	0·65	1·05 (0·57-1·93)	0·90	··	··	··	··	··	··
MB$_{\text{Group4-HR}}$	37/78 (47%)	0·33 (0·20-0·54)	0·00023	0·58 (0·36-0·93)	0·13	0·41 (0·25-0·68)	0·0043	0·33 (0·18-0·61)	0·00038	··	··	··	··
I *	7/90 (8%)	··	··	··	··	··	··	··	··	··	··	··	··
II	11/90 (12%)	1·72 (0·90-3·29)	0·26	4·08 (1·92-8·67)	0·0063	2·43 (1·22-4·84)	0·058	··	··	5·32 (2·18-12·99)	0·00023	··	··
III	13/90 (14%)	4·07 (2·10-7·86)	0·00020	1·32 (0·70-2·48)	0·60	1·89 (1·00-3·60)	0·18	3·20 (1·43-7·13)	0·0045	··	··	··	··
IV *	2/90 (2%)	··	··	··	··	··	··	··	··	··	··	··	··
V	11/90 (12%)	0·91 (0·46-1·79)	0·99	1·74 (0·90-3·38)	0·19	1·24 (0·64-2·37)	0·69	··	··	··	··	··	··
VI *	7/90 (8%)	··	··	··	··	··	··	··	··	··	··	··	··
VII	12/90 (13%)	0·81 (0·44-1·52)	0·86	0·56 (0·29-1·09)	0·18	0·71 (0·37-1·36)	0·53	··	··	··	··	··	··
VIII	27/90 (30%)	0·57 (0·35-0·92)	0·081	0·62 (0·37-1·04)	0·18	0·57 (0·34-0·96)	0·15	··	··	··	··	··	··

HR=hazard ratio. MB=medulloblastoma. *=variable not included in analyses due to infrequent events.

Supplementary Table 5: Univariable and multivariable survival analyses of correlates of time-based measures in the MB$_{\text{Group3}}$ and MB$_{\text{Group4}}$ irradiated group

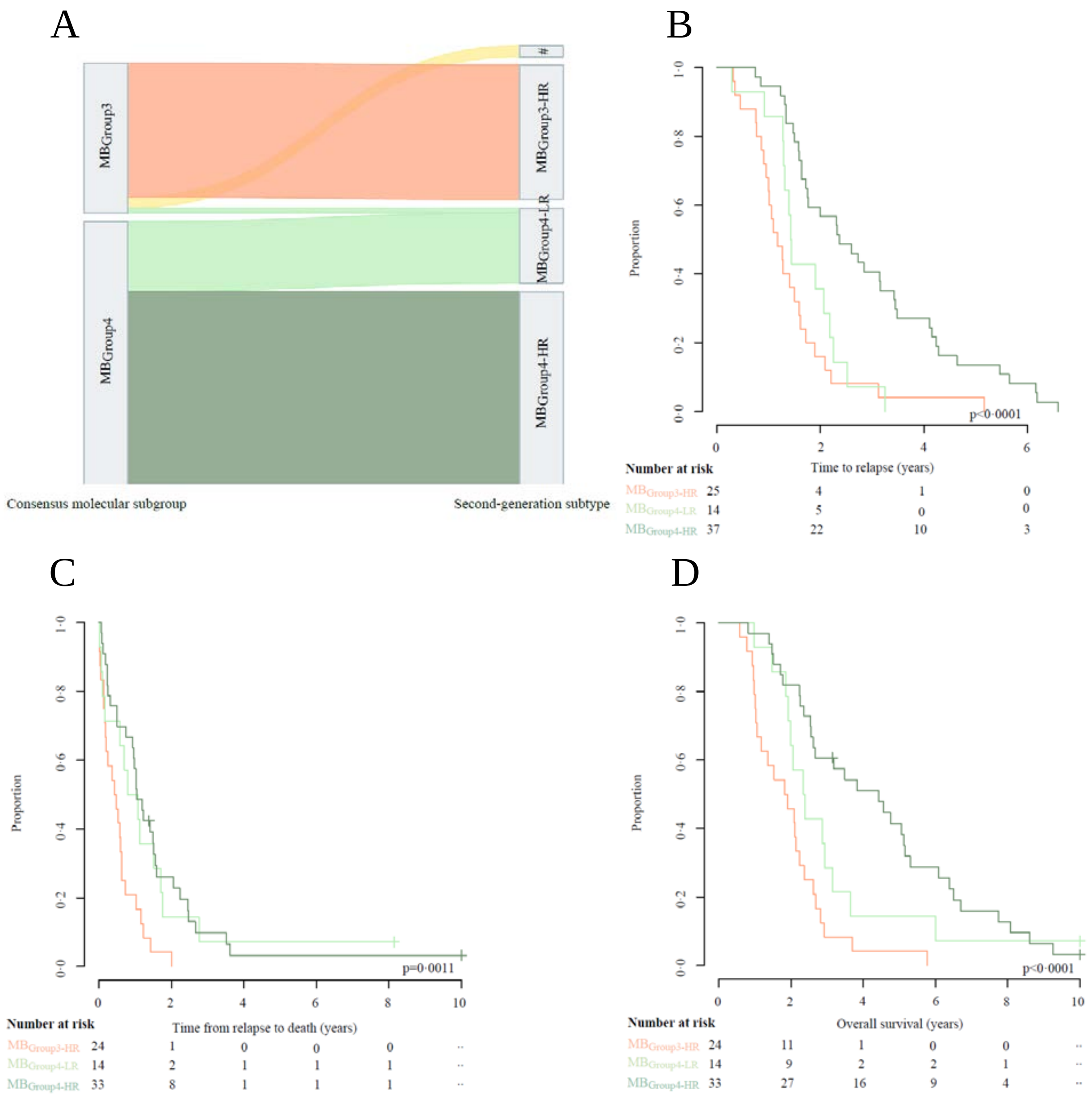

E

	Distant disease at diagnosis*	Distant disease at relapse†	p value#	Acquired distant disease$
$MB_{Group3\text{-}HR}$	10/25 (40%)	17/18 (94%)	0·00030	12/13 (92%)
$MB_{Group4\text{-}LR}$	7/14 (50%)	8/9 (89%)	0·086	4/5 (80%)
$MB_{Group4\text{-}HR}$	12/36 (33%)	31/33 (94%)	<0·0001	21/22 (95%)

*No significant difference by molecular subtype p=0.55. †No significant difference by molecular subtype p=0.84. #p value comparing the proportion of distant disease at diagnosis versus distant disease at relapse within MB_{Group3} and MB_{Group4} second-generation subtypes. $No significant difference by molecular subtype p=0.50.

F

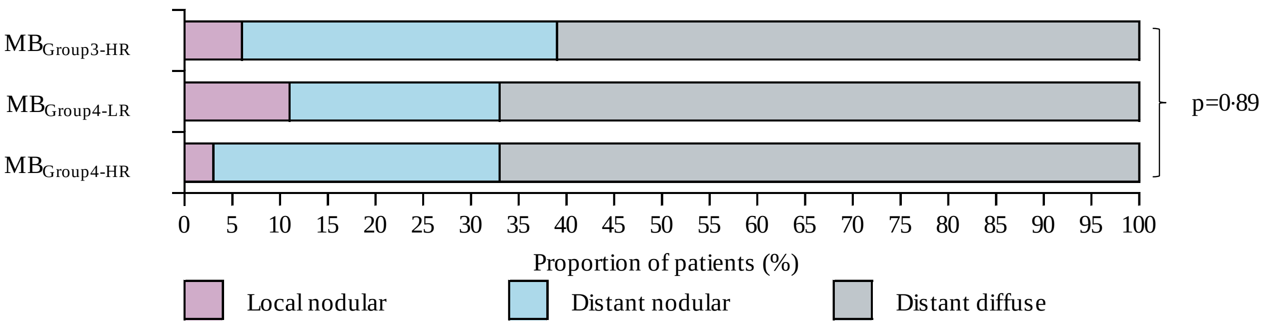

Supplementary Figure 6: Assessment of relapse characteristics by second generation MB_{Group3} and MB_{Group4} subtypes. (A) Sankey plot of the relationship between consensus MB_{Group3} and MB_{Group4} and second-generation subtypes. (B) Time to relapse according to second-generation MB_{Group3} and MB_{Group4} molecular subtype. (C) Time from relapse to death according to second-generation MB_{Group3} and MB_{Group4} molecular subtype. (D) Overall survival according to second-generation MB_{Group3} and MB_{Group4} molecular subtype. (E) The prevalence of distant disease at diagnosis and distant disease acquired at relapse according to second-generation MB_{Group3} and MB_{Group4} molecular subtype. (F) The different patterns of relapsed disease according to second-generation MB_{Group3} and MB_{Group4} molecular subtype. $MB_{Group3-HR}$=18 patients, $MB_{Group4-LR}$=9 patients, $MB_{Group4-HR}$=30 patients. MB=medulloblastoma. #=MB_{Group3} low-risk. Only MB_{Group3} and MB_{Group4} subtypes present in >10% of cohort included in analyses.

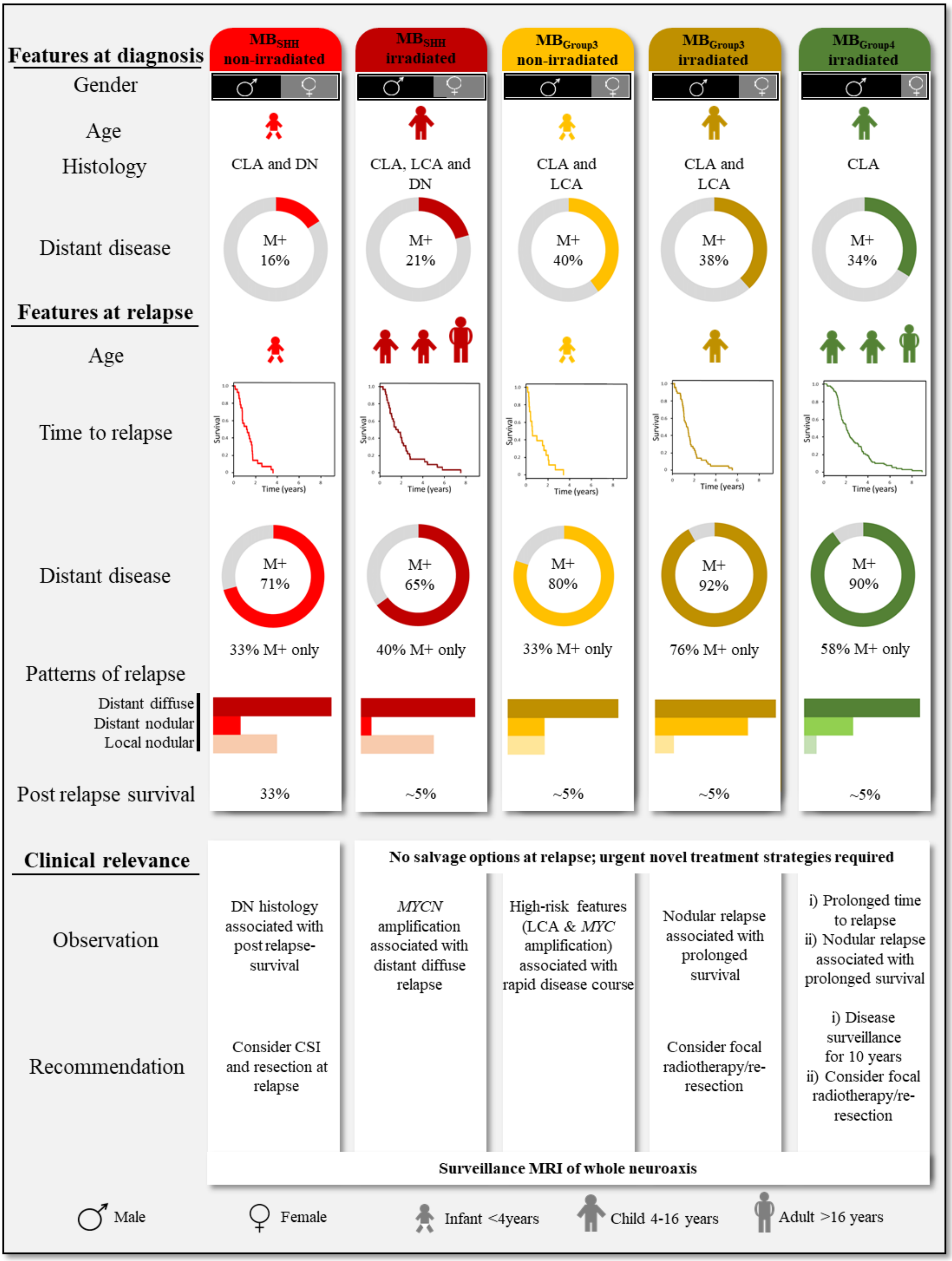

Supplementary Figure 7: Pattern and timing of medulloblastoma relapse in our cohort according to upfront treatment and molecular subgroup: clinical relevance and recommendations. MB=medulloblastoma. SHH=sonic hedgehog. CSI=cranio-spinal irradiation. CLA=classic histology. DN=desmoplastic or nodular histology. LCA=large-cell anaplastic histology. M+=distant disease. MRI=Magnetic Resonance Imaging.
